# Antimicrobial activity of a novel Spanish propolis against planktonic and sessile oral *Streptococcus* spp

**DOI:** 10.1038/s41598-021-03202-1

**Published:** 2021-12-13

**Authors:** M. Luisa Navarro-Pérez, Virginia Vadillo-Rodríguez, Irene Fernández-Babiano, Ciro Pérez-Giraldo, M. Coronada Fernández-Calderón

**Affiliations:** 1 Department of Biomedical Science, Area of Microbiology, University of Extremadura, Badajoz, Spain; 2 Instituto Universitario de Investigación Biosanitaria de Extremadura (INUBE), Badajoz, Spain; 3 Department of Applied Physics, Area of Applied Physics, University of Extremadura, Badajoz, Spain; 4 Biomedical Research Network Centre on Bioengineering, Biomaterials and Nanomedicine (CIBER-BBN), Badajoz, Spain

**Keywords:** Microbiology, Health care

## Abstract

Increased bacterial resistance to traditional antimicrobial agents has prompted the use of natural products with antimicrobial properties such as propolis, extensively employed since ancient times. However, the chemical composition of propolis extracts is extremely complex and has been shown to vary depending on the region and season of collection, due to variations in the flora from which the pharmacological substances are obtained, being therefore essential for their antimicrobial activity to be checked before use. For this purpose, we evaluate the in vitro antimicrobial and anti-biofilm activity of a new and promising Spanish ethanolic extract of propolis (SEEP) on *Streptococcus mutans* and *Streptococcus sanguinis*, responsible, as dominant ‘pioneer’ species, for dental plaque. Results reveal that *S. sanguinis* is more sensitive to SEEP, slowing and retarding its growth considerably with lower concentrations than those needed to produce the same effect in *S. mutans*. SEEP presents concentration- and time-dependent killing activity and, furthermore, some of the subinhibitory concentrations employed increased biofilm formation even when bacterial growth decreased. Mono and dual-species biofilms were also inhibited by SEEP. Findings obtained clearly show the relevance of using biofilm and subinhibitory concentration models to determine optimal treatment concentrations.

## Introduction

Propolis is a natural complex resinous mixture obtained from beehives, produced by honeybees mixing products collected from tree buds, plants, saps, resins, and other botanical sources, with beeswax and salivary enzymes^1^. Since ancient times, propolis has been extensively used, especially in folk medicine, to treat various maladies^2^. In the last decades, investigation of its constituents and biological properties has gained increasing attention^3,4^.

Increased bacterial resistance to traditional antimicrobial agents and its effects has prompted the use of natural products with antimicrobial activity such as propolis^5,6^. The low toxicity of propolis has made it a good candidate to act as adjuvant in the treatment or prevention of many infectious diseases^7^.In particular, propolis has been shown to be a promising cariostatic agent, although due to variations in its chemical composition further studies are needed to establish quality and safety control criteria, i.e., by analyzing its chemical profile to determine the proportion of chemical compounds and to be able to ensure the safety of these compounds^8^.

The chemical composition of propolis depends on the phytogeographic characteristics of the site of collection, since bees choose different plants as a source of propolis in different habitats^9^. The new Spanish ethanolic extract of propolis (SEEP) used in this research contains high amounts of polyphenols, with unusually more than half of these in the flavonoid class, and some of the unique compounds found in this Mediterranen-type propolis (Vanillic acid, 1-Acetoxypinoresinol, p-HPEA-EA and 3,4-DHPEA-EDA) have also been identified in olive oil which are widely known to have antimicrobial activities and other health benefits^10^. In addition, SEEP shows antibacterial activity against *Staphylococcus epidermidis*^10^ and antifungal activity against *Candida glabrata*^11^.

Based on their excellent biological properties, the use of propolis extract is considered for the prevention and treatment of oral diseases, associated to the accumulation of pathogenic biofilm. The oral cavity is one of the most complex and populated microbial niches in the human body^12^. Several hundred different microorganisms are part of the microbiota and mostly coexist within the biofilm that constitutes dental plaque^13^. Several studies indicate streptococci as the dominant ‘pioneer’ species^14^. The genus *Streptococcus* represents a high percentage of all supragingival microorganisms present in the oral biofilm. In this sense, *S. mutans* is one of the most common members of the Mutans group and plays an important role in the etiology of human dental caries and peri-implant infections^15^. For its part, *S. sanguinis* belongs to the most abundant of the oral streptococci found within the human oral cavity and, despite being opportunistic pathogens, they are typically associated with healthy plaque biofilm^16^. Interspecies interactions are possibly mediated through a well-regulated production of chemicals and could play an essential part in balancing competition/coexistence within multispecies microbial communities^17^. Current studies suggest that *S. sanguinis* competes with *S. mutans*, which can lessen or prevent dental caries. However, as a pioneering colonizer, *S. sanguinis* may also enable attachment of succeeding pathogens facilitating biofilm formation^16^.

The structural characteristics of biofilms give them different properties compared with planktonic cells. According to previous studies, a biofilm can withstand the defensive acts of the host better than planktonic cells and has a higher resistance to antibiotics^18,19^. Recalcitrant and persistent biofilm-associated diseases have raised the need for new therapeutic approaches and methods for reliably culturing mature biofilms and evaluating their chemical, structural, and physiological characteristics^20^. In addition, natural antimicrobial compounds like propolis are currently being widely consumed in as everyday products such as chewing gums, toothpaste, or oral sprays. Continuous exposure to subinhibitory concentrations could have an impact on the development of resistance. For this reason, it is important to carry out studies to clarify the possible effects of each concentration used.

The present study focuses on the use of SEEP for the prevention and treatment of oral diseases. It evaluates in, particular, the antibacterial and anti-biofilm activity of this new SEEP against two pioneer Gram-positive colonizers of oral cavity, *Streptococcus mutans* and *Streptococcus sanguinis*.

## Results and discussion

### Antibacterial testing

The minimal inhibitory concentration (MIC) and minimal bactericidal concentration (MBC) values of SEEP estimated for *S. mutans* were 240 µg/mL (0.4%) and 480 µg/mL (0.8%), respectively. For *S. sanguinis*, the measured MIC and MBC values were lower, i.e., 60 µg/mL (0.1%) and 120 µg/mL (0.2%), respectively. The biological activity of SEEP detected was not influenced by the presence of ethanol in the propolis solutions, as a minimum solvent concentration of around 12.5% was required to inhibit the growth of both strains. MIC and MBC values of Ampicillin sodium (positive control) for *S. mutans* were 0.16 µg/mL and 0.31 µg/mL, respectively, and 0.08 µg/mL and 0.31 µg/mL for *S. sanguinis*, values close to those already published^21,22^.

It is difficult to compare MIC and MBC results from different studies due to the chemical variability of the propolis samples employed and/or the different methods used for their evaluation. Nevertheless, an increase in the bactericidal concentration to eradicate the growth of *S. mutans* with regard to MIC was also observed with propolis from other regions such as Argentina, MIC: 50 µg/mL, MBC: 460 µg/mL^23^, Brasil, MIC: 293 μg/mL, MBC: 1172 µg/mL^24^, MIC: 50 μg/mL, MBC: 50 µg/mL^25^, Poland, MIC: 39–156 μg/mL, MBC: 313–1250 µg/mL^26^, among others.

### Growth kinetics

**Figure 1:**
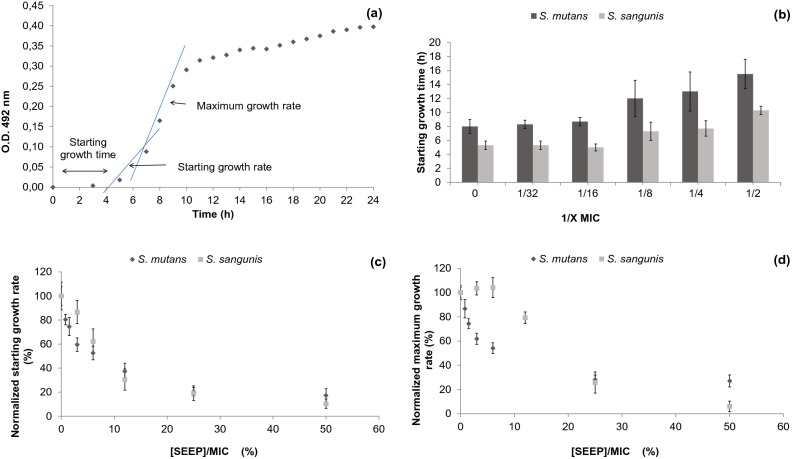
(**a**) Representation of a typical bacterial growth curve highlighting the parameters analysed. (**b**) The time in hours (mean ± SD) that both strains need to start growth. (**c**) Normalized starting slope of exponential growth and (**d**) normalized exponential growth slope (mean ± SD) of *S. mutans* and *S. sanguinis*.

The growth curves obtained were described by three key parameters: starting growth time, starting growth rate and maximum growth rate (Fig. 1a), all of which were found to be affected by the presence of SEEP.

The control culture starting growth time points of *S. mutans* and *S. sanguinis* lasted approximately 8 h and 5 h, respectively (Fig. 1b). In both cases, a delay in time to start growth was observed when using concentrations between 1/2 and 1/8 subinhibitory concentrations (sub-MICs). At 1/2 MIC, both strains needed twice the time taken by the controls to begin to grow. The starting and exponential growth rates were normalized with respect to the control values in order to better compare the two strains. As can be seen in Fig. 1c, the starting growth rate of both strains appears equally affected by sub-MICs, decreasing exponentially with the increase of the concentration of propolis relative to their MICs. In terms the maximum growth rate (Fig. 1d), however, sub-MICs affected both strains differently. In this latter case, the rate of *S. mutans* was found to decline from the beginning, whereas the *S. sanguinis* rate maintained similar values to that of the control until a SEEP concentration of 1/16 MIC was reached. From this concentration on, the maximum growth rate of *S. sanguinis* decreased faster than it did for *S. mutans*, and finally reached slightly lower values at the highest SEEP concentration studied. Sub-MIC concentrations therefore delay the onset and initial growth rate of both strains equally, but they affect both strains differently once the exponential growth phase is reached. Importantly, this later observation could potentially be useful for the design of optimal dosage regimes and suggests that determination of the typically reported MIC and specific maximum growth rate values alone is probably incomplete.

Sub-MICs can delay the onset of the lag phase and slow down the exponential phase once it has begun. The slowdown effect on *S. mutans* growth was also observed with natural honey in a study in which potential antibacterial properties were examined^27^. This feature may be considered as an advantageous property for dental plaque control in the early stages of biofilm formation. Indeed, different studies have already proposed propolis as a treatment of dental caries and mouth infections^28^, e.g., as a component in toothpaste oral cavity^29,30^. In any case, before it can be recommended for routine application in dentistry clinics or in the manufacture of oral products for home use, guidelines for quality control of this natural product should be developed^8^.

### Lethality curves assay

Time-to-kill assay is a kinetic method of determining whether bacterial killing is concentration and/or time dependent, i.e., it assesses the ability to kill in relation to time and with different fixed concentrations of antimicrobial agents^31^.

**Figure 2:**
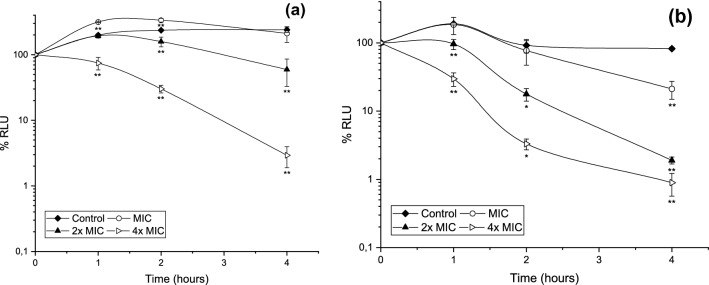
Time-kill curves for (**a**) *S. mutans* and (**b**) *S. sanguinis* in exponential growth exposed to different SEEP concentrations during 1, 2 and 4 h of contact. Values represented as mean ± SD. RLU, relative luminescent units. Statistical analysis: n = 4/treatment/hours; significant difference versus Control (* 0.01 ≤ *P* ≤ 0.05; ** *P* < 0.01) by one-way ANOVA (Dunnett’s or Tukey’s post-test).

Figure 2 shows the evolution of control cultures growing exponentially (100% of relative luminescent units, RLU) after being placed in contact with propolis for different periods of time with different concentrations of SEEP. These data show that *S. mutans* control cultures grew during the first four hours analysed. Those cultures in contact with MICs (Fig. 2), and sub-MICs (data not shown), began to decrease after two hours of contact, although their metabolic activity up to that point was statistically higher than that of the controls (*P* < 0.01). At 2 × MIC bacterial growth was only observed in the first hour of contact and, after four hours, growth was significantly reduced by more than half (*P* < 0.01). The most effective propolis concentration was 4 × MIC (0.96 mg/mL), which inhibited growth from the moment of contact (*P* < 0.01) and produced a significant bactericidal effect after 4 h. A similar study on optimal concentrations of Korean propolis against isolates of mutans streptococci revealed a bacteriostatic effect on *S. mutans* ATCC 25175 at 4 × MIC (0.14 mg/mL) after 24 h^32^, but given the lack of standardization of extraction methods and in vitro tests, interpretation of results such as this is complicated^8^.

In the case of *S. sanguinis,* the most effective propolis concentration was also 4 × MIC (0.24 mg/mL), which significantly inhibits growth from the first hour of contact (*P* < 0.01), i.e., the bactericidal effect was observed earlier. In this case, there was no significant increase in growth in the first hours of contact with MICs and sub-MICs. At 24 h of growth, all propolis concentrations tested showed no growth.

Time-kill, therefore, was concentration and exposure time dependent. Experiments on bacterial killing kinetics of SEEP using planktonic organisms have shown that the extracts might act differentially in the tested strains. SEEP acts faster and in lower concentrations against an exponentially growing culture of *S. sanguinis* as compared to *S. mutans*. As reported by^33^, this experimental observation suggests that only concentrations greater than or equal to 2 × MIC of SEEP inhibit bacterial metabolic reactions that precede cell doubling.

### Influence of subinhibitory concentrations on biofilm bacterial formation

**Figure 3:**
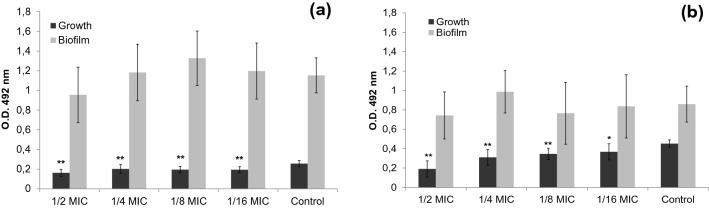
Bacterial growth, biofilm formation (mean ± SD) after 24 h of incubation of (**a**) *S. mutans* and (**b**) *S. sanguinis* with different sub-MICs of SEEP. Error bars show the mean ± SD. *S. mutans*: n = 18–20/treatment, *S. sanguinis*: n = 14/treatment; significant difference to Control values (* 0.01 ≤ *P* ≤ 0.05; ** *P* < 0.01) by one-way ANOVA (Tukey's test).

The values of growth and biofilm formation obtained after 24 h in the presence of the different sub-MICs of propolis studied are shown in Fig. 3. Both strains show a similar pattern of growth and biofilm formation concerning the control inocula. In particular, it was found that growth of the treated cells was significantly lower than that of the control cultures. Biofilm formation, on the other hand, did not show significant differences with respect to the control values.

However, when the *Slime Index* (SI) is considered according to Eq. (1), which represents the relationship between bacterial growth and biofilm formation, it is found that the values obtained show a significant difference with regard to the last two sub-MICs compared to the control (100%) in the case of *S. mutans*: 1/8 MIC = 151%**, 1/16 MIC = 137%*, while in the case of *S. sanguinis* only the first two sub-MICs showed significant differences: 1/2 MIC = 204%**, 1/4 MIC = 167%** (* 0.01 ≤ *P* ≤ 0.05 and ** *P* < 0.01). This means that the increase in biofilm formation is higher than expected based on the bacterial growth produced. This increase is caused by lower propolis sub-MICs in the case of *S. mutans* and by higher ones in the case of *S. sanguinis*.

**Figure 4:**
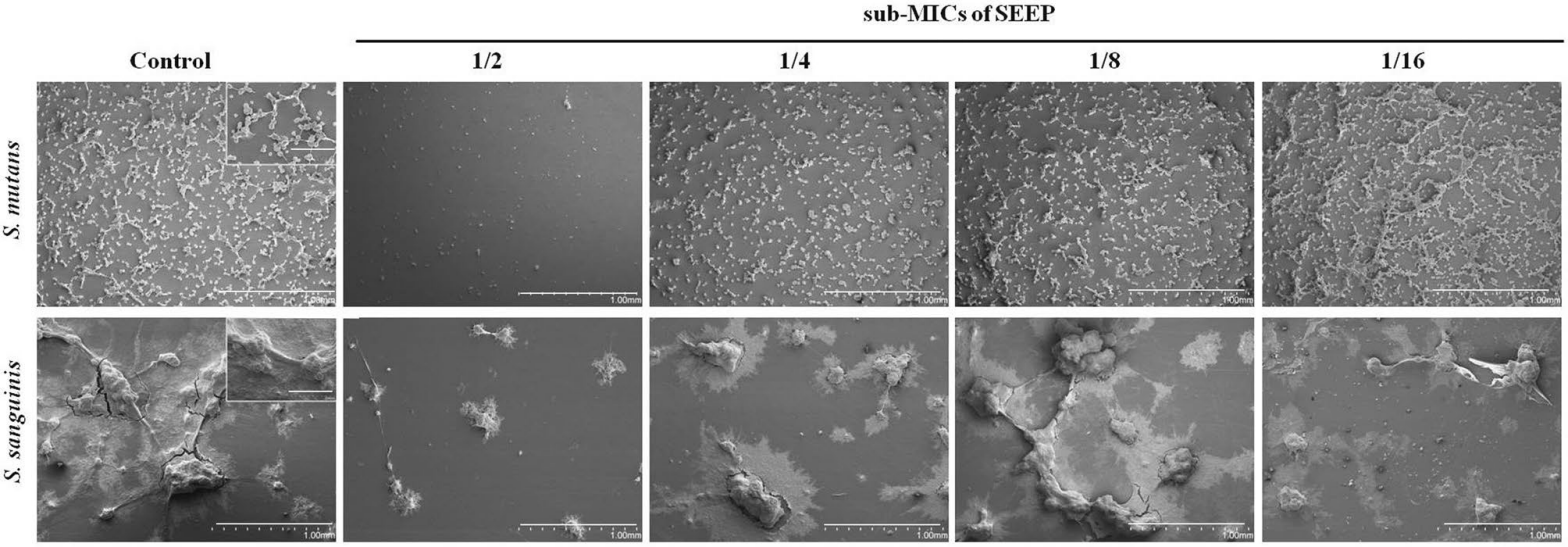
Representative SEM images of biofilms untreated (control) and SEEP-treated with respective sub-MICs against *S. mutans* and *S. sanguinis*. The images magnification was 50 × and 100 µm scale bars. The images inserted in the control ones have a magnification of 250 × and 200 µm scale bars.

Figure 4 shows representative SEM images of biofilms after 24 h of untreated (control) and SEEP-treated cells. It is observed that *S. sanguinis* produces large clusters of bacteria that could cause an increase in the slime index at higher MICs (1/2 × and 1/4 × MIC), as previously mentioned. *S. mutans* biofilm does not show such large bacterial clusters, it is more homogeneously distributed and, at the same time, there is a higher amount of bacteria in the 1/8 × and 1/16 × MIC. Previous studies on coaggregating and non-coaggregating oral bacterial have highlighted the involvement of positive cooperativity in the coaggregation process due to streptococci, preferably binded to a coaggregate rather than to available free different bacteria^34^. Future studies in this respect should be carried out to consider the differences found in the structure of the biofilms produced by the strains here studied.

The sub-MICs of some antiseptic compounds, such as chlorhexidine, sodium fluoride^35^ or triclosan^36^ are known to increase the expression of genes related to *S. mutans* biofilm formation. This observation has also been demonstrated for other related species, such as for *Streptococcus pyogenes* treated with fluoroquinolones^37^ or *Streptococcus sobrinus* treated with several antibiotics^38^. However, it has been shown that natural compounds such as emodin^39^, tea polyphenols^35^, leaves of *Dodonaea viscosa* var. *angustifolia*^40^ or farnesol^41^ can decrease *S. mutans* biofilm at sub-MICs, but no previous studies have analysed the relationship between growth and biofilm formation (expressed as Slime Index, SI) for these two *Streptococcus* strains. The present study demonstrates that some sub-inhibitory concentrations, also of propolis, can increase biofilm formation even when bacterial growth has decreased.

### Activity on mature biofilms

**Figure 5:**
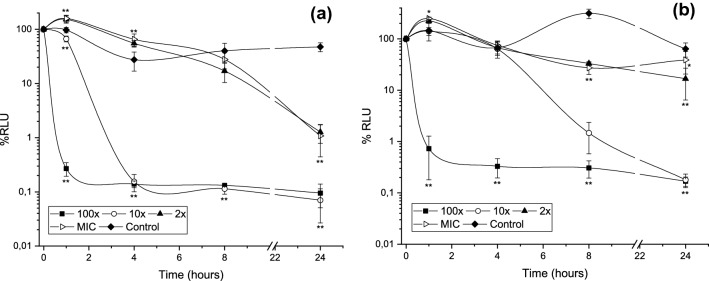
Percentage of metabolic bacterial activity of mature biofilms of (**a**) *S. mutans* and (**b**) *S. sanguinis* treated with different SEEP concentrations during different hours of contact. Values represented as mean ± SD. RLU, relative luminescent units. Statistical analysis: n = 6–10/treatment/hours; significant difference versus Control (* 0.01 ≤ *P* ≤ 0.05; ** *P* < 0.01) by one-way ANOVA (Dunnett’s post-test).

The influence of SEEP on monobacteria and dual-species mature biofilms is shown on Fig. 5 and Table 1, respectively. The propolis extract at 100 × MIC (*S. mutans*: 24 mg/mL and *S. sanguinis*: 6 mg/mL) was the most significantly (P < 0.01) effective concentration, substantially decreasing the metabolic bacterial activity of the biofilms in 1 h, the shortest time tested (Fig. 5a). *S. mutans* mature biofilm was also significantly (*P* < 0.01) eradicated with 10 × MIC (2.4 mg/mL) after 4 h of contact; however, 10 × MIC (0.6 mg/mL) against *S. sanguinis* needed at least 8 h of contact with SEEP to significantly (*P* < 0.01) eliminate its mature biofilm. As in the time-to-kill assay with the exponentially growing bacteria, in monobacteria mature biofilm a significant increase in activity was also observed after the first hours of contact with the lowest concentrations studied (*S. mutans*: *P* < 0.01; *S. sanguinis* 0.01 ≤ *P* ≤ 0.05). These low concentrations may initially stimulate bacterial activity, although after some time they are able to decrease viability in the biofilms.

**Table 1:** Percentage of viable sessile cells of mature mixed biofilms (mean ± SD) relative to the control at time zero (100%) after different hours of contact with different concentrations of SEEP.

Hours	Control	0.06 mg/ml (1)	0.12 mg/ml (2)	0.24 mg/ml (3)	0.48 mg/ml (4)	0.6 mg/ml (5)	2.4 mg/ml (6)	6 mg/ml (7)	24 mg/ml (8)
1	100.9 ± 25.7	113.8 ± 23.4	107 ± 27.3	93.4 ± 29.4	81.7 ± 23.9	68.1 ± 21.9	31 ± 17.7*	1.9 ± 1**	0.3 ± 0**
4	141.9 ± 60.4	81.1 ± 9.8	72.4 ± 15.9	77.2 ± 32.1*	67 ± 31**	50.7 ± 22.3**	0.5 ± 0.2**	0.4 ± 0.1**	0.3 ± 0.1**
8	177.3 ± 59.9	100.1 ± 16.2	49.8 ± 11.2**	33.1 ± 8.3**	13.1 ± 2.5**	8.1 ± 2.3**	0.3 ± 0**	0.3 ± 0.1**	0.2 ± 0**
24	169.8 ± 56.5	5.4 ± 1.9**	1 ± 0.3**	0.3 ± 0.1**	0.3 ± 0.1**	0.5 ± 0.3**	0.1 ± 0**	0.1 ± 0**	0.1 ± 0**

(1) MIC of SEEP against *S. sanguinis*; (2) SEEP concentration 2 × MIC against *S. sanguinis*;(3) MIC of SEEP against *S. mutans*; (4) SEEP concentration 2 × MIC against *S. mutans*;(5) SEEP concentration 10 × MIC against *S. sanguinis*; (6) SEEP concentration 10 × MIC against *S. mutans*;(7) SEEP concentration 100 × MIC against *S. sanguinis*; (8) SEEP concentration 100 × MIC against *S. mutans*.

* 0.01 ≤ *P* ≤ 0.05.

**P < 0.01.

It seems, therefore, that 4 h of contact with propolis is sufficient to totally inhibit activity of *S. mutans* (see Fig. 5a). Specifically, 0.96 mg/mL (4 × MIC) is needed to inhibit activity of planktonic bacteria on exponential growth (see results on “Lethality curves assay”), and 2.4 mg/mL (10 × MIC) to inhibit activity of bacteria already included in a mature biofilm. Duarte et al.^42^, however, evaluating activity of a Brazilian propolis extract against a *S. mutans* UA159 5-day-old biofilm found that 0.8 mg/ml acting during 4 h did not have any major killing activity against this older biofilm, although acid production of the biofilms was notably reduced.

In the case of *S. sanguinis*, the lowest concentration tested that inhibited activity of planktonic bacteria was 0.12 mg/mL (2 × MIC) after 4 h of contact (see Fig. 5b), while the mature biofilm needed 0.6 mg/mL for 8 h to be eliminated (see, Fig. 5b). Such concentrations may be considered harmless to humans when applied in the oral cavity^43^. Furthermore, the Spanish propolis extract studied for these experiments does not contain toxic metal compounds^10^, which could cause propolis toxicity^44^. However, special care should be taken with the use of lower concentrations as results have shown an increase in metabolic activity of both sessile and planktonic bacteria during the first hours of contact.

The viability of dual-species biofilm treated with SEEP differs from that of single-species biofilms in that the former begins to be significantly affected already with the MIC of *S. mutans* (Table 1). Nevertheless, the lowest concentration required to totally inhibit dual-species mature biofilms in the shortest possible contact time was the same as that for the biofilm of the most resistant strain, *S. mutans*, i.e., 2.4 mg/mL (10 × MIC) of SEEP during 4 h of contact. Furthermore, in this case, no significant increase in viability was observed with the lower concentrations studied. Thus, the results indicate that SEEP also inhibited dual-species biofilm activity. Due to the antagonistic relationship of *S. mutans* and *S. sanguinis* within a biofilm^16,17^, further bacterial composition analyses in dual-species biofilms are necessary to evaluate the proportion of each strain when both coexist in the same biofilm that is treated with and without an antimicrobial compound^45^.

The combination of mechanisms for the observed ability of SEEP to reduce biofilm formation and to removal mature biofilms is unknown. It has been proposed that the diterpenes and triterpenes of Mediterranean propolis exhibit very strong antimicrobial activity against *S. mutans*^46^, such as flavonoids of Anatolian propolis ^47^, benzophenones of Brazilian geopropolis^48,49^ or Apigenin and tt-farnesol also identified in Brazilian and Chilean propolis^50–52^. SEEP is a Mediterranean-type propolis that does not have the typical pattern of “poplar type” propolis. Its composition is characterized by a high amount of polyphenol (205 ± 34 mg GAE/g), flavonoids (127 ± 19 mg QE/g), and phenolic compounds found in extra virgin olive oil such as vanillic acid, 1-Acetoxypinoresinol, p-HPEA-EA and 3,4-DHPEA-EDA, never before detected in propolis samples^10^. Previously, the interaction of SEEP and/or its molecules with bacterial cells has been studied from a physicochemical approach. In such studies, it has been detected that propolis induces substantial changes in volumetric charge density, electrophoretic smoothness and degree of hydrophobicity of the external surface layer of the cells (both gram-positive and gram-negative). According to the results of this research, the proposed mechanism of action of SEEP against bacteria appears to be initially physical, producing structural damage to the membrane/wall^53^. Afrasiabi et al.^54^ also observed scattering of the *S. mutans* biofilm structure by propolis nanoparticles, which can may cause the loss of membrane potential, required for bacterial viability.

Future studies of SEEP compounds separately will help to better understand the mechanism of action against oral biofilm formation, and thus help to avoid the defensive effect produced by the natural extract at sub-inhibitory concentrations. Since nutrients can come from dead bacterial cells, inefficient SEEP concentrations could lead to adhesion and proliferation of the surviving bacteria^55^. As other authors note^8^, using such purified compounds from propolis would be easier to standardize and would provide a selective pressure which is generally associated with the emergence of bacterial resistance.

The studies carried out to date concerning SEEP and its potential action against the in vitro oral bacteria studied here guarantee the effectiveness of this natural product and contribute to the continuation of pharmacokinetic and pharmacodynamic investigations^56^ which will test the efficacy in vivo of this natural substance.

## Conclusions

The following conclusions can be drawn from the present study:

- The Spanish propolis is effective against oral *Streptococcus* strains, *S. mutans* and *S. sanguinis*, responsible, as dominant ‘pioneer’ species, for dental plaque.
- *S. sanguinis* is more susceptible to SEEP, as it slows and retards its growth considerably with lower concentrations than those necessary to cause the same effect in *S. mutans*.
- It is the first time that the relationship between growth and biofilm formation, expressed as *Slime Index*, has been analysed for these two *Streptococcus* strains. In particular, this study demonstrates that some of the SEEP sub-inhibitory concentrations employed increase biofilm formation to the detriment of bacterial growth. In addition, SEM images reveal significant clustering among the *S. sanguinis* cells, while *S. mutans* yields relatively homogeneous biofilms.
- Mono and dual-species biofilm are inhibited by this ancestral compound and findings obtained clearly show the relevance of using biofilm models and sub-MICs to determine antibacterial activity and their optimal treatment concentrations of the tested extract.
- Further studies are required to isolate the most active molecules of SEEP against these and other oral bacteria and to elucidate their mechanisms of action, with a perspective for their use either alone or as an adjuvant in the treatment of oral infections.

## Materials and methods

### Preparation of Spanish ethanolic extract of propolis (SEEP)

The SEEP and its solvent (70% ethanol) were sterilized with filters of 0.45 µm (Millipore, Merck, Germany) and stored at 4 ºC until use. Serial dilutions were made in Trypticase Soy Broth (TSB, BBL™ BD, Becton, Dickinson and Company, Spark, NV, USA) supplemented with 1% of filter-sterilized sucrose (PANREAC, AppliChem GmbH—An ITW Company, Ottoweg, Darmstadt. Germany), hereinafter TSBs, to obtain final concentrations of 12.5% to 0.025%, which correspond with 7.688 mg to 0.015 mg of dry weight of propolis per milliliter^10^.

### Bacterial strains and growth conditions

The strains used in the present study, i.e., *S. mutans* ATCC 25175 and *S. sanguinis* ATCC 10556, come from The American Type Culture Collection (ATCC). The strains were inoculated in blood agar plates (OXOID LTD., Basingstoke, Hampshire, UK) and incubated at 37 °C in an incubator with 5% of CO_2_ (New Brunswick™ Galaxy® 170S, Eppendorf AG, Hamburg, Deutschland, Germany) to obtain cultures. Subsequently, they were cultivated in Brain Heart Infusion Agar plates (BHA) (OXOID LTD., Basingstoke, Hampshire, UK) under the same conditions mentioned above to refresh colonies.

Both species were routinely grown overnight at 37 °C  and 5% CO_2_ in TSBs. After this time, each bacterial suspension was adjusted to 82% of transmittance, equivalent to 0.086 of optical density (O.D.), at 492 nm wavelength. A spectrophotometer (Helios epsilon Model, Thermiospectronic, Waltham, MA, USA) was used for the measurement and subsequently, the suspension was diluted 1/100 or 1/10 in TSBs to obtain approximately 10^6^ or 10^7^ CFU/mL, respectively and thus to be used as inocula in the remaining experiments.

### Antibacterial activity of SEEP

**Minimal Inhibitory Concentration** (MIC) against *S. mutans* and *S. sanguinis* strains were determined by microdilution methodology in accordance with the Clinical and Laboratory Standards Institute (CLSI) guidelines^57^. Serial dilution tests, with different propolis extract concentrations in sterile TSBs, were performed in 96-well polystyrene flat-bottomed microtiter plates (Greiner bio-one, Frickenhausen, Germany). The wells, containing 100 μL of the different concentrations of SEEP studied and 100 μL of the bacterial suspensions (10^6^ CFU/mL) were incubated with 5% CO_2_ for 24 h at 37 °C. The MIC value was defined as the lowest concentration of SEEP in which no visual growth was found (O.D. ≤ 0.09) and was expressed in µg/mL. Growth was measured as optical density (O.D.) at 492 nm with a microplate spectrophotometer reader (ELx800; Bio-Tek Instruments, Inc. Winooski, VT, USA). The O.D. of the wells containing SEEP without bacterial cells was subtracted from those with bacterial growth in order to rule out interferences due to the colour of propolis for each of the SEEP concentrations investigated, thus, values below 0.09 were considered as no bacterial growth. **Minimal Bactericidal Concentration** (MBC) was determined on agar plates (BHA) by incubating 20 µl of the previously cultured wells for 24 h at 37 °C and 5% CO_2_. The MBC was the lowest concentration that precluded bacterial growth on the agar plates. Three separate experiments in duplicate were conducted for each concentration of SEEP and its solvent. Negative controls, without bacteria, and positive, with Ampicillin sodium from 25 to 0.02 µg/mL (Oxoid, Ireland), were also tested.

### Growth kinetics

Bacterial growth curves of *S. mutans* and *S. sangunis* were obtained by turbidity measurements. Specifically, cells were cultivated, with and without propolis, as described above in 96-well microtitre plates, and the optical density of each of the samples was recorded at regular intervals from zero time to 24 h. Once determined, growth curves were described by the following parameters: starting growth time, starting growth rate and maximum growth rate (Fig. 1a). The first parameter describes the time at which the absorbance value first differs significantly from zero. After this point in time, cells were found to experience a gradual continuous growth before entering the exponential phase. The slope of these data points, i.e., those preceding the exponential phase, was estimated and defined as the starting growth rate. Finally, maximum growth rate is defined as the highest speed of growth detected along the growth curve and corresponds to the slope of the curve at the exponential phase. The data presented represent the mean ± SD of at least three-independent curves obtained from different cultures.

### Lethality curves assay

Time-to-kill assays were performed in 96-well microtiter plates containing 100 μL of approximately 10^6^ CFU/mL cultures that were grown for 12 h to reach the beginning of stationary grown phase. At this point, before adding the different SEEP concentrations, the first measure of metabolic activity of bacteria (zero time) was made. The procedure to measure bacterial killing was by ATP bioluminescence assay which allows determination of the number of viable microbial cells in cultures based on the quantification of adenosine triphosphate, which is a chemical form of energy of all living cells, measured by a luminometer and expressed as relative luminescent unit^58^.

To carry out the following measurements, 50 μL of each well was replaced, taking care not to cause any contamination, with 50 μL of fresh medium with and without SEEP. After 1, 2, 4 and 24 h of contact, viability measurements were performed by adding 100 μL of the reagent BacTiter-Glo™ (Promega Corporation, Madison, WI, USA) to the wells and allowed to interact for 5 min in the dark. Finally, the contents were transferred to 96-well white polystyrene flat-bottomed microtiter plates (Greiner Bio-One, Frickenhausen, Germany) and light emission (luciferin-luciferase reaction) was measured in a fluorescence microplate reader (FLx800; Bio-Tek Instruments, Inc. Winooski, VT, USA). In this assay, the surfaces without bacteria were included as negative controls and propolis-free medium served as positive controls. The experiments were carried out at least three times with independent cultures in order to confirm reproducibility.

### Anti-biofilm activity of SEEP

Biofilm formation was performed in 96-well microtiter plates. The initial inoculum was approximately 10^6^ CFU/mL and was added to the wells containing 100 µl of TSBs with and without propolis. Positive and negative controls were also included as described above. Bacterial growth was measured after 24 h of incubation as O.D. at 492 nm using a spectrophotometer ELx800. Each well was aspirated, and samples were carefully washed twice with phosphate buffered saline (PBS) using a suction pump (Model FTA-2i, Biosan SIA. Riga, Latvia). The biofilms adhered to the bottom of the wells were heat-fixed in a Pasteur Heraeus electronic oven (C.R. Maré, S.A., Barcelona, Spain) for at least 4 h at 60 °C, and stained with violet Violet Crystal (VC, Gram-Hucker DC; Panreac, Barcelona, Spain) for 5 min. The excess dye was removed with water. The plates were dried and later the dye bound to the adhered bacteria was resuspended with 200 μL of glacial acetic acid (GAA, Fisher Scientific, Loughborough, UK) for 10 min. The optical density of the plates was again measured by a microplate reader at 492 nm. Each assay was performed in triplicate and repeated at least three times.

The results were analysed by Eq. (1): *Slime Index* (SI) according to Pérez-Giraldo et al.^59^ with modifications. SI evaluates whether the biofilm O.D. value was related to the corresponding value in O.D. of bacterial growth. The formula applied was SI% = 100 × [(mean O.D. of biofilm treated with SEEP / mean O.D. of growth treated with SEEP) / (mean O.D. of biofilm control / mean O.D. of growth control)].

### Activity of SEEP on mature biofilms

Mature biofilms were obtained from inocula (200 μL; 10^7^ CFU/mL) of *S. mutans*, *S. sanguinis* and mixed cultures incubated at 37 °C for 24 h in a 96-well white polystyrene flat-bottomed microtiter plates. The 1-day-old biofilm was washed twice with sterile PBS using a suction pump to remove nonadherent cells. These biofilms, established for 24 h, were subsequently treated with different propolis concentrations and the control with TSBs. After incubation during different contact times, viability of bacteria in biofilm was measured by bioluminescence reaction^60^. First, the supernatant was removed and washed twice with PBS to remove non-adherent bacteria, and then 200 μL of BacTiter-Glo™ reagent prepared according to the manufacturer’s instructions was added to each well. Results were expressed as percentages relative to control at zero time without SEEP treatment.

### Scanning electron microscopy (SEM)

Bacterial suspensions were incubated at 37 °C for 24 h (5% CO_2_) with TSBs and TSBs containing the different subinhibitory concentrations of SEEP studied on glass coverslips circles within 12-well microtitre plates (BioLite 12 Well Multidish, Thermo Fisher Scientific, Rochester, NY. USA). After 1, 4, 8 and 24 h of incubation, the covers were carefully washed twice with sterile TSBs to eliminate the non-adherent bacteria.

The growth biofilms were fixed at room temperature with 3% vol/vol glutaraldehyde (PanreacQuímica SAU, Barcelona, Spain) for approximately 15 h and dehydrated in a series of ethanol solutions (30, 50, 70, 90 and 100% vol/vol) for 1 h each. The samples were then dried in a vacuum chamber, coated with a thin layer of gold (≤ 5 nm) using an EMITECH K575K (Quorum Technologies Ltd., West Sussex, UK) sputter coater, and finally, the image was captured with a Scanning Electron Microscope (HITACHI S-4800, Hitachi High-Technologie, Tokyo, Japan).

### Statistical analysis

Data are presented as mean and standard deviation is determined across the duplicates with at least three independent experiments. Statistical analyses were performed using SPSS 22.0 (SPSS Inc., Chicago, IL, USA). In all experiments, to compare multiple means of treated samples versus control a one-way analysis of variance test (ANOVA) followed by Tukey's post hoc test or by Dunnett's T3 post hoc test when Levene's test revealed unequal variance. When the data were not normally distributed, the Kruskal–Wallis and multiple-comparison tests were used to compare values.

## Data Availability

Correspondence and request form datasets generated and analysed during the current study should be addressed to MLNP and MCFC.
